# Galectins‐1 and ‐3 in Human Intervertebral Disc Degeneration: Non‐Uniform Distribution Profiles and Activation of Disease Markers Involving NF‐κB by Galectin‐1

**DOI:** 10.1002/jor.24351

**Published:** 2019-06-24

**Authors:** Mahmoud Elshamly, Katharina Kinslechner, Josef G. Grohs, Daniela Weinmann, Sonja M. Walzer, Reinhard Windhager, Hans‐Joachim Gabius, Stefan Toegel

**Affiliations:** ^1^ Department of Orthopedics and Trauma Surgery, Karl Chiari Lab for Orthopaedic Biology Medical University of Vienna 1090 Vienna Austria; ^2^ Department of Orthopedics and Trauma Surgery, Division of Orthopedics Medical University of Vienna 1090 Vienna Austria; ^3^ Institute of Physiological Chemistry, Faculty of Veterinary Medicine Ludwig‐Maximilians University Munich, 80539 Munich Germany; ^4^ Ludwig Boltzmann Institute for Arthritis and Rehabilitation Vienna Austria

**Keywords:** anulus fibrosus, degeneration, lectin, notochord, nucleus pulposus

## Abstract

Degeneration of the human intervertebral disc (IVD) is assumed to underlie severe clinical symptoms, in particular chronic back pain. Since adhesion/growth‐regulatory galectins are linked to arthritis/osteoarthritis pathogenesis by activating a pro‐degradative/‐inflammatory gene expression signature, we hypothesized a similar functional involvement of galectins in IVD degeneration. Immunohistochemical evidence for the presence of galectins‐1 and ‐3 in IVD is provided comparatively for specimens of spondylochondrosis, spondylolisthesis, and spinal deformity. Immunopositivity was detected in sections of fixed IVD specimens in each cellular compartment with age‐, disease‐, and galectin‐type‐related differences. Of note, presence of both galectins correlated with IVD degeneration, whereas correlation with age was seen only for galectin‐3. In addition, staining profiles for these two galectins showed different distribution patterns in serial sections, an indication for non‐redundant functionalities. In vitro, both galectins bound to IVD cells in a glycan‐dependent manner. However, exclusively galectin‐1 binding triggered a significant induction of functional disease markers (i.e., IL6, CXCL8, and MMP1/3/13) with involvement of the nuclear factor‐kB pathway. This study thus gives direction to further network analyses and functional studies on galectins in IVD degeneration. © 2019 The Authors. *Journal of Orthopaedic Research*® published by Wiley Periodicals, Inc. on behalf of Orthopaedic Research Society. J Orthop Res 37:2204–2216, 2019

## INTRODUCTION

Low back pain ranks among the most common medical complaints, placing an enormous burden on the individual patient, with broad‐scale socioeconomic implications for our society.^1^, ^2^ Although the underlying routes toward its manifestation appear complex and are not yet precisely defined, the well‐documented link to degeneration of the lumbar intervertebral disc (IVD) warrants a hypothesis‐driven study to relate histopathological changes to molecular characteristics.^3^

Obviously, identifying effectors of degeneration has potential to provide therapeutic perspective, as novel targets can initiate the development of innovative treatment modalities. In this context, it is noteworthy that cells of the nucleus pulposus (NP) have been likened to articular chondrocytes.^4^ Being hereby guided to look at tissue degeneration in osteoarthritis, we previously found that an emerging group of elicitors of functional disease markers in cartilage degeneration belongs to the class of endogenous lectins. In particular, we recently revealed the upregulation of distinct endogenous galectins in osteoarthritic cartilage^5^ and their involvement in triggering a pro‐degradative/‐inflammatory microenvironment via expression of nuclear factor‐kB (NF‐kB)‐regulated gene expression profiles.^6^, ^7^, ^8^ Mechanistically, tissue lectins “read” cellular signals encoded by glycans and translate their information into molecular activities.^9^, ^10^ In osteoarthritis, members of the galectin family, that is, galectins‐1, ‐3, and ‐8 (Gal‐1, Gal‐3, and Gal‐8), serve as signaling‐inducing mediators (for a recent systematic literature review on galectins in (osteo)arthritis, see Salamanna et al.^11^). The potential of clinical relevance in cartilage and joint degradation has led to call Gal‐3 “a key player in arthritis”.^12^ Consequently, these proteins are receiving increasing attention, aiming to detect new connections to disease mechanisms in auto‐immune regulation and beyond.^13^, ^14^ This current line of investigation has prompted us to assume that galectins may also play a functional role in IVD degeneration. Indeed, the occurrence of two galectins has already been documented for IVD cells in development as well as in healthy and degenerated tissue specimens.

During embryogenesis, Gal‐3 is found in the mammalian notochord (with species‐specific timing) and later in the future vertebrae, while Gal‐1 expression is observed in IVDs.^15^, ^16^, ^17^, ^18^ Thus, Gal‐3—alone or in combination with CD24 and carbonic anhydrase 12—has acquired the status of an NP cell marker.^19^, ^20^, ^21^, ^22^, ^23^ Gal‐1 presence was reported in human, porcine and rat IVDs in NP and anulus fibrosus (AF) cells, in which its distribution pattern was similar to that of the matrix glycoprotein laminin (isoform LM‐511),^24^, ^25^ a known counterreceptor of Gal‐1 and ‐3.^26^, ^27^ Functionally, Gal‐3 presence has been assumed to affect NP cell survival^28^ or “the destructive potential” of NP cells.^21^ Regulation of its expression in rat NP cells by hypoxia‐inducible factor‐1α^28^ or by transforming growth factor‐β through canonical Smad3 signaling underscores its potential pathophysiological significance, as does its synergy with tumor necrosis factor‐α on increasing levels of interleukin‐1β (IL‐1β), IL‐6, and chemokine CCL2 gene expression^29^ factors relevant for inflammation and disc degeneration.^30^, ^31^

This study assessed three groups of clinical specimens for expression status of Gal‐1 and ‐3, to test the hypothesis of a role of galectins in IVD degeneration. In vitro, IVD cells were additionally examined for (i) secretion of galectins, (ii) carbohydrate‐inhibitable galectin binding, (iii) ensuing stimulation of transcription and secretion of selected functional disease markers and, if positive, (iv) an influence of galectins on NF‐κB‐dependent signaling.

## MATERIALS AND METHODS

### Galectins and Antibodies

Recombinant proteins, AlexaFluor‐labeled galectins, and non‐crossreactive antibody preparations against Gal‐1 or −3 were prepared and applied as previously described in detail.^6^, ^7^, ^32^

### Clinical Specimens and Data

The study was approved by the ethics committee of the Medical University of Vienna (EK‐No.: 1720/2015). Surgical IVD specimens were obtained with written consent from patients treated routinely with transforaminal lumbar interbody fusion. Only specimens that contained all three major anatomical parts of the IVD (i.e., AF, NP, and endplate [EP]), hereby allowing histological scoring (as described below), were included in the study. The medical background of the patients covering age, sex, and diagnosis was documented. In addition, magnetic resonance imaging (MRI), computer tomography, and X‐ray data of the patients were available, and the degree of radiological degeneration was assessed using the Pfirrmann classification.^33^ Clinical data enabled assignment of patients into three study groups, that is, spondylochondrosis, true spondylolisthesis, and spinal deformity (idiopathic scoliosis and/or kyphosis). Further details on clinical specimens are given in Method section in Supplementary Material.

### Histological Assessment

IVD specimens were fixed in formalin and decalcified using Titriplex‐Tris‐solution, then dehydrated and embedded in paraffin according to the standard procedures. Paraffin sections (2.5 μm) were stained with hematoxylin and eosin (HE; for morphological evaluation) or Safranin O (SO; for evaluation of glycosaminoglycan content), counterstained using light green Goldner III solution. The degree of degeneration was graded by microscopic evaluation of the sections according to an established histological scoring system with minor modifications.^34^ In brief, presentation of the major anatomical structures of the IVD (i.e., EP, AF, AF/NP boundaries, NP cells, and matrix) as well as IVD staining with SO were included in the analysis. In each subcategory, the level of degeneration was scored as 0, 1, or 2, based on defined histological characteristics.^34^ Summing up the six individual scores, a total histological score of degeneration was computed, ranging from 0 (intact IVD) to 12 (most strongly degenerated IVD). The histological grading was performed independently by two observers. Cases of deviating assessments were discussed to reach agreement.

### Immunohistochemistry

Immunohistochemical processing and semiquantitative analysis followed a standardized protocol using non‐cross‐reactive antibody preparations.^5^, ^32^

Percentage of immunopositivity (i.e., staining of cell nucleus, cytoplasm and pericellular matrix as well as the extracellular matrix in the case of the NP) was determined, and staining profiles in each region were graded from 1 to 4 based on the labeling index (LI): LI = 0 (0% positive cells or matrix), 1 (1–25%), 2 (26–50%), 3 (51–75%), and 4 (76–100%). Summing up the LIs in the five regions resulted in a total LI, ranging from 0 (absence of staining) to 20 (most extensive presence of positivity).

### Isolation and Culture of IVD Cells

Primary human IVD cells were isolated following published protocols.^35^ In brief, IVD specimens were obtained from eight patients (11 spinal levels, age 32–70 years, five female, three male) undergoing spinal surgery due to spondylochondrosis (*n* = 7 patients) or spondylolisthesis (*n* = 1). Disc tissues were separated from EPs, rinsed with phosphate‐buffered saline (PBS) and cut into small pieces, which were treated enzymatically using a 0.2% (w/v) solution of collagenase overnight at 37°C. IVD cells were cultured thereafter in growth medium consisting of Dulbecco's modified Eagle's medium supplemented with 10% fetal calf serum, penicillin (50 units/ml), streptomycin (50 μg/ml), and amphotericin B (25 μg/ml). Cultures were kept at 37°C in a humidified atmosphere with 5% CO_2_ and used for experiments at passage 1. Following overnight starvation, cells were exposed—in absence or presence of 40 µM CAPE (Merck, Darmstadt, Germany)—for 24 h to 10 µg/ml Gal‐1, 18 µg/ml Gal‐3 or with a mixture thereof prior to analysis using quantitative reverse‐transcription polymerase chain reaction (RT‐qPCR). For western blot experiments, cells were incubated with 10 µg/ml Gal‐1 for 15 min.

### Detection of Galectin‐Binding Sites on the Surface of IVD Cells

Following previously established protocols,^7^ cultured IVD cells (*n* = 4 patients) were harvested by trypsinization, and a cell suspension of 3 × 10^5^ cells in 50 μl PBS was incubated with a mixture of AlexaFluor555‐labeled Gal‐1 (1 μg/50 μl) and AlexaFluor488‐labeled Gal‐3 (2 μg/50 μl) for 10 min at 4°C, in the presence or absence of 0.1 M lactose to control for inhibition by cognate glycan. Images were immediately taken without fixation using laser scanning microscopy (Carl Zeiss, Oberkochen, Germany; LSM700; Zen software).

### RT‐qPCR

Isolation of total RNA, complementary DNA synthesis, and SYBR‐green‐based qPCR experiments were performed as previously described.^5^, ^36^ A detailed checklist containing all relevant information^37^ is provided in Supplementary Table S1. Messenger RNA (mRNA) levels were calculated as relative quantities compared to the untreated controls considering amplification efficiencies and normalization to succinate dehydrogenase complex, subunit A (SDHA).

### Enzyme‐Linked Immunosorbent Assays (ELISAs)

The levels of pro‐MMP‐1, pro‐MMP‐13, and total‐MMP‐3 were detected in cell culture supernatants of Gal‐1‐ or Gal‐3‐treated IVD cells (all ELISAs; R&D Systems, Minneapolis, MN). Supernatants of untreated IVD cells served as controls. Also, supernatants of untreated IVD cells were processed for galectin secretion (ELISAs from R&D Systems). Ranges of standard curve were 0.313–20 ng/ml for Gal‐1, 0.157–10 ng/ml for Gal‐3, 0.157–10 ng/ml for pro‐MMP‐1 and total‐MMP‐3, and 78–5,000 pg/ml for pro‐MMP‐13.

### Western Blot

Western blot analyses were performed as previously described.^8^ Briefly, membranes (nitrocellulose blotting membrane 0.2 µm; GE Healthcare Life Sciences, Freiburg, Germany) were incubated for 2 h with primary antibodies specific for phospho NF‐κB p65 (Ser536; 1:1,000; rabbit monoclonal; Cell Signaling, Danvers, MA), NF‐kB p65 (1:1,000; mouse monoclonal; Cell Signaling), and β‐actin (1:5,000; mouse monoclonal; Cell Signaling). Thereafter, membranes were incubated for 1 h with a solution containing IRDye 800CW goat anti‐rabbit IgG (1:15,000; LI‐COR, Bad Homburg, Germany) and IRDye 680LT goat anti‐mouse IgG (1:15,000; LI‐COR, Bad Homburg, Germany). Signal intensities were quantified using the Odyssey Imager CLx (LI‐COR, Bad Homburg, Germany). The ratios between levels of phospho‐p65 and total p65 (both normalized to β‐actin) were calculated and depicted as absolute signal intensities.

### Statistics

Data were analyzed using IBM (Armonk, NY) SPSS v25 with descriptive statistics, parametric, and non‐parametric inferential statistics, as well as Spearman correlation analyses, where *r* values were interpreted as follows: 0–0.2: weak correlation, >0.2–0.4: mild correlation, >0.4–0.6: moderate correlation, >0.6–0.8: moderately strong correlation, and >0.8–1: strong correlation. Kruskal–Wallis test (with pairwise comparison) was used for comparing the differences in median values of certain parameters between the disease groups, while Friedman test (with pairwise comparison) was used to compare the median values of different parameters within the same group. The qPCR data were analyzed using Wilcoxon or Friedman tests with pairwise comparison. Significance values were adjusted by Bonferroni correction for multiple comparisons.

## RESULTS

### Clinical and Histological Classification of IVD Specimens

After having rigorously tested the clinical material regarding its suitability for histological scoring, specimens from 23 patients with spondylochondrosis, eight patients with spondylolisthesis, and seven patients with spinal deformity could be included into this study. Details on the patients’ age, sex, and the Pfirrmann grades of IVDs are given in Table 1. To document the morphological status, exemplary T2‐weighted MRI data from representative patients of each group are presented in Supplementary Figure S1a.

**Table 1:** Demographics and Patients’ Characteristics

	Spondylochondrosis	Spondylolisthesis	Deformity	Kruskal–Wallis test
Number (female/male)	*n* = 23	*n* = 8	*n* = 7	
	F = 16/M = 7	F = 6/M = 2	F = 3/M = 4	
Levels	L2–3 (*n* = 3)	L5‐S1 (*n* = 8)	Th10‐11 (*n* = 1)	
	L3–4 (*n* = 4)		Th11‐12 (*n* = 1)	
	L4–5 (*n* = 8)		L3–4 (*n* = 1)	
	L5‐S1 (*n* = 8)		L4–5 (*n* = 1)	
			L5‐S1 (*n* = 3)	
Age (years; mean ± SD)	58.8 ± 11.4	37.4 ± 9.4	33.2 ± 19.7	0.005^a^
				0.02^b^
				>0.05^c^
Pfirrmann score (median ± IQ range)	4 ± 1	3 ± 3	1.5 ± 1	>0.05^a^
				0.001^b^
				>0.05^c^

Shown are the *p* values of Kruskal–Wallis test for age and the Pfirrmann score between the three cohorts:

^a^ Spondylochondrosis versus spondylolisthesis.

^b^ Spondylochondrosis versus deformity.

^c^ Spondylolisthesis versus deformity.

Supplementary Figure S1b shows histological IVD sections from representative specimens of each of the three cohorts stained with HE or SO. Comparison of total histological scores revealed that spondylochondrosis specimens had significantly higher median scores than spondylolisthesis or deformity specimens (*p *< 0.05; Supplementary Figure S1c). As shown in Supplementary Table S2, histological alterations in all six histological subcategories contributed to a significant difference between spondylochondrosis and either spondylolisthesis or deformity specimens.

When all data were combined, a moderately strong correlation (*r *= 0.783, *p *< 0.0001) between histological score and Pfirrmann grade was found (Supplementary Figure S1d). Taken together, the analyzed IVD specimens provided a solid basis for immunohistochemical analyses.

### Immunohistochemical Localization of Gal‐1 in Degenerated IVD

**Figure 1:**
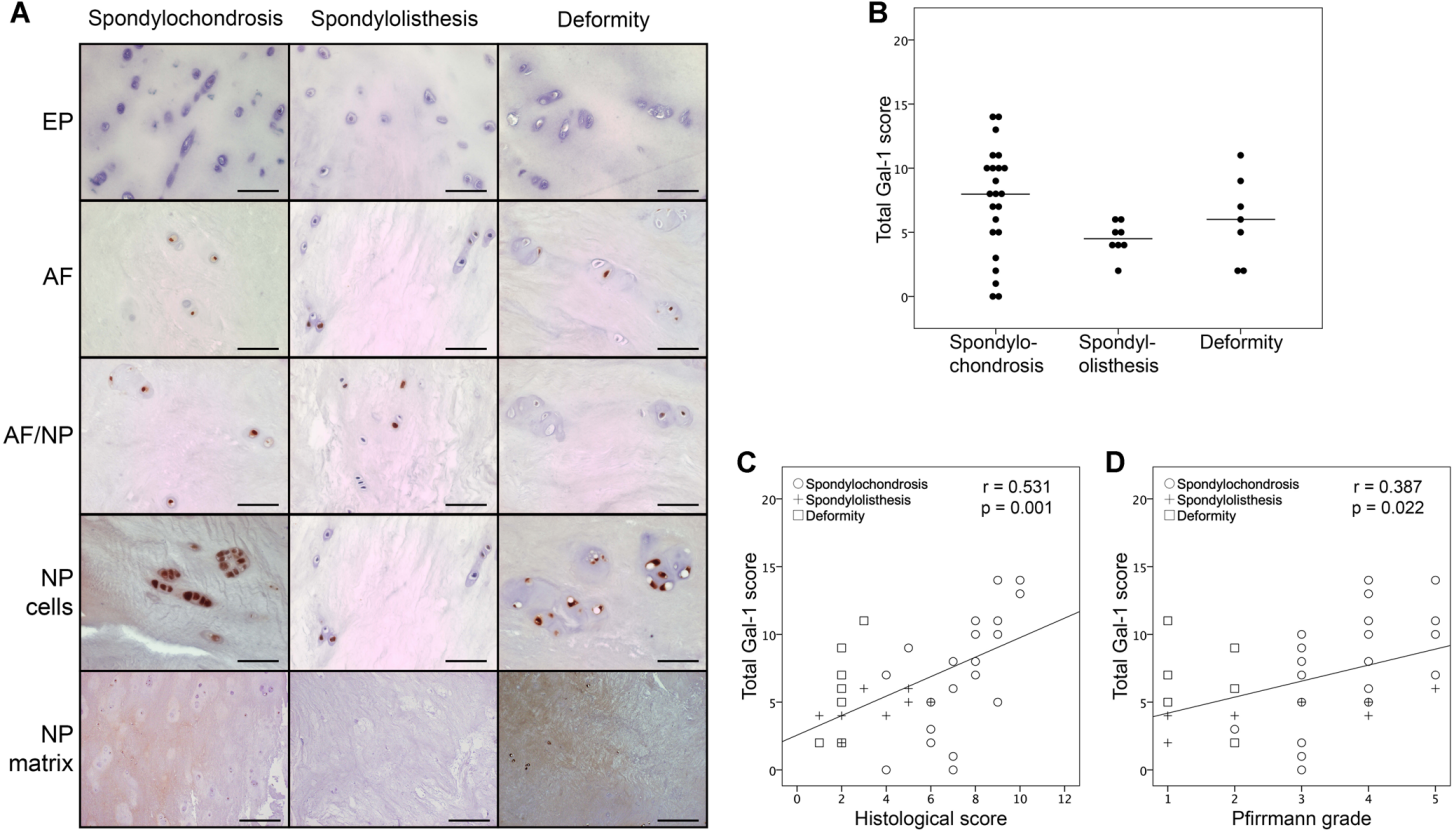
Immunohistochemically detected presence of Gal‐1 in clinical intervertebral disc (IVD) specimens and its correlation with clinical and histological signs of degeneration. (A) Shown are representative IVD specimens from patients of the spondylochondrosis (left column), the spondylolisthesis (middle column), and the deformity cohorts (right column). The Gal‐1 positivity (brown color) among cells in the endplate (EP), the anulus fibrosus (AF), the boundary region between AF and NP (AF/NP), as well as for cells or matrix of the nucleus pulposus (NP) is documented. Scale bars = 50 or 200 µm (NP matrix). (B) Comparison of total Gal‐1 LI scores between patients of the spondylochondrosis, spondylolisthesis, and deformity cohorts. Results are presented as dotplots showing Gal‐1 LI scores for patients of each cohort. The median values are indicated as bars. *p < 0.05 (Kruskal–Wallis test). (C,D) Shown are scatterplots of total Gal‐1 LI scores versus (C) histological IVD scores or (D) Pfirrmann grades for patients of all three cohorts with the regression line. The Spearman correlation coefficient *r* and the *p* value (bivariate correlation test) were calculated given in this panel. [Color figure can be viewed at wileyonlinelibrary.com]

**Figure 2:**
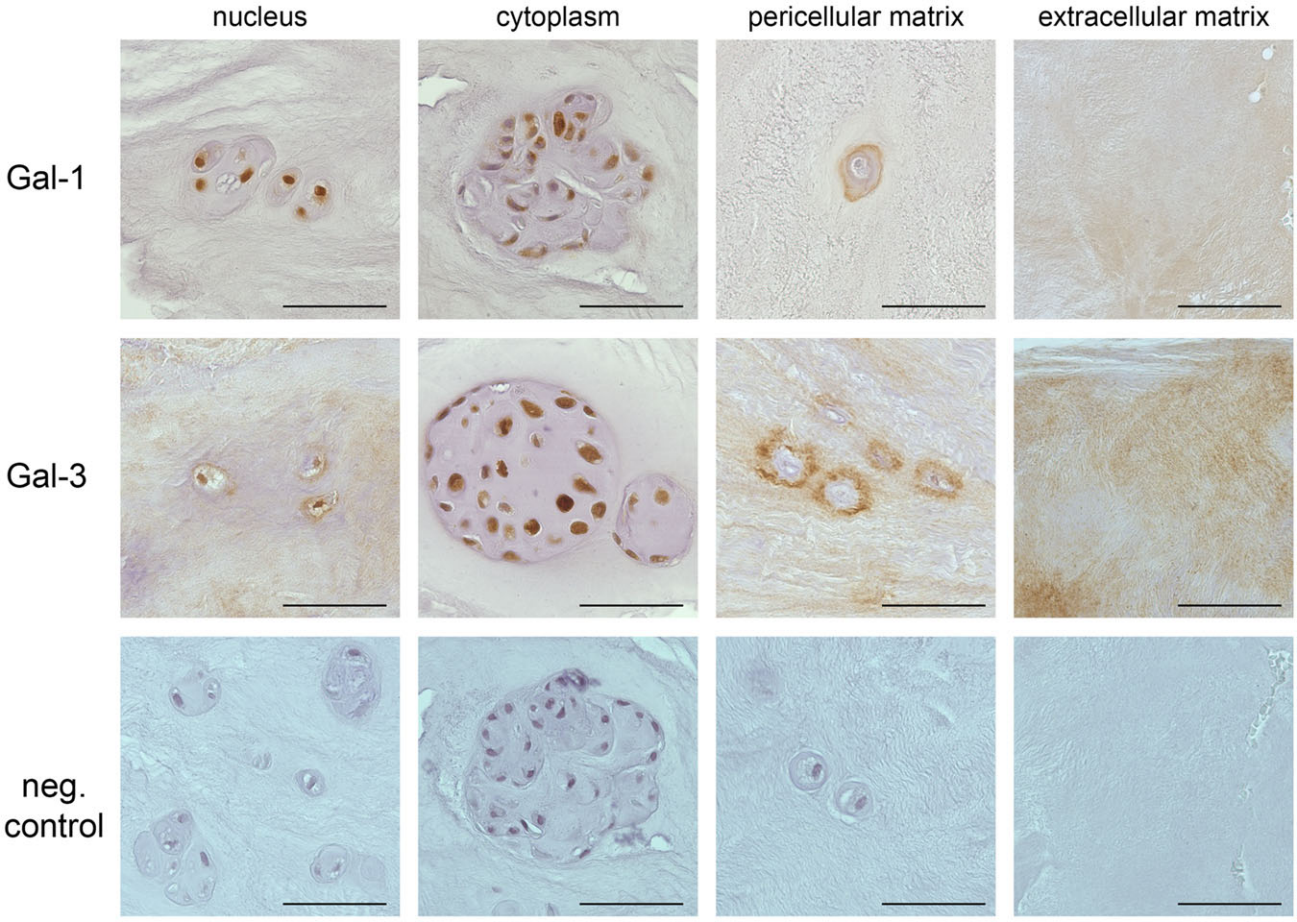
Immunohistochemically detected presence of Gal‐1 and Gal‐3 in clinical intervertebral disc (IVD) specimens at different sites, that is, nuclear and cytoplasmic positivity and staining of pericellular and extracellular matrix. Negative controls in absence of primary antibodies are shown for all sites. Scale bars = 50 µm. [Color figure can be viewed at wileyonlinelibrary.com]

The percentages of Gal‐1 positivity in EP, AF, cells, or matrix of the NP as well as the AF/NP boundary were determined microscopically. Figure 1A shows staining for Gal‐1 in histological IVD sections from representative patients. Nuclei, cytoplasm (particularly in cells of large chondrons) as well as pericellular and the extracellular matrix were immunopositive (Fig. 2).

The median total Gal‐1 LI scores (±IQ range) were 8 ± 5 in the spondylochondrosis cohort, 4.5 ± 1.75 in the spondylolisthesis cohort and 6 ± 7 in the deformity cohort. Within the spondylochondrosis specimens, the extent of Gal‐1 positivity was significantly higher in cells of the AF and the NP than in cells of the EP (*p* = 0.01 and *p *= 0.001, respectively; Table 2). Within the spondylolisthesis specimens, level of Gal‐1 positivity was significantly higher in cells of the AF/NP boundary region than in cells of the EP (*p *= 0.01; Table 2), whereas it was significantly higher in NP cells than in EP cells in deformity specimens (*p* = 0.01; Table 2).

**Table 2:** Immunohistochemical Scores of Gal‐1 Positivity

	Spondylochondrosis (Median ± IQR)	Spondylolisthesis (Median ± IQR)	Deformity (Median ± IQR)	Kruskal–Wallis Test	Overall Median ± IQR
EP	0 ± 1	0 ± 0	0 ± 0	>0.05^a^	0 ± 1
				>0.05^b^	
				>0.05^c^	
AF	2 ± 2	1 ± 0	1 ± 2	>0.05^a^	1 ± 1
				>0.05^b^	
				>0.05^c^	
AF/NP	2 ± 3	2 ± 1	1 ± 2	>0.05^a^	1.5 ± 1
				>0.05^b^	
				>0.05^c^	
NP cells	3 ± 2	1 ± 1.75	2 ± 2	>0.05^a^	2 ± 2
				>0.05^b^	
				>0.05^c^	
NP matrix	1 ± 2	0 ± 1	1 ± 3	>0.05^a^	0.5 ± 2
				>0.05^b^	
				>0.05^c^	
Total Gal‐1 score	8 ± 5	4.5 ± 1.75	6 ± 7	>0.05^a^	6 ± 6
				>0.05^b^	
				>0.05^c^	
Friedman test (pairwise comparison)	*p* = 0.01 (EP vs. AF)	*p* = 0.01 (EP vs. AF/NP)	*p* = 0.01 (EP vs. NP cells)		*p *< 0.0001 (EP vs. AF/NP)
	*p* = 0.001 (EP vs. NP cells)				*p* < 0.0001 (EP vs. AF)
					*p* < 0.0001 (EP vs. NP cells)
					*p *= 0.007 (NP matrix vs. NP cells)

AF, anulus fibrosus; EP, endplate; IQR, interquartile range; IVD, intervertebral disc; NP, nucleus pulposus.

Shown are the median values (±IQR) of Gal‐1 positivity scores in EP, AF, AF/NP, and NP cells and in NP matrix as well as the total Gal‐1 positivity scores for all three cohorts. In addition, *p* values of Kruskal–Wallis test between the three cohorts as well as *p* values of the Friedman test (with pairwise comparison and Bonferroni correction) between the different components of the IVD within a cohort are presented.

^a^ Spondylochondrosis versus spondylolisthesis.

^b^ Spondylochondrosis versus deformity.

^c^ Spondylolisthesis versus deformity.

Comparison of total Gal‐1 LI scores revealed no statistically significant difference between the three cohorts (*p* > 0.05; Fig. 1B and Table 2). Table 2 further shows that the three study groups did not significantly differ in Gal‐1 LI scores in subcategories.

Correlation analyses of all specimens, irrespective of the assigned study group, revealed a moderate correlation between total Gal‐1 LI and histological scores (*r* = 0.531, *p *< 0.001; Fig. 1C) and a mild correlation between total Gal‐1 LI score and Pfirrmann grade (*r* = 0.387, *p *= 0.022; Fig. 1D).

### 3.3 Immunohistochemical Localization of Gal‐3 in the Degenerated IVD

**Figure 3:**
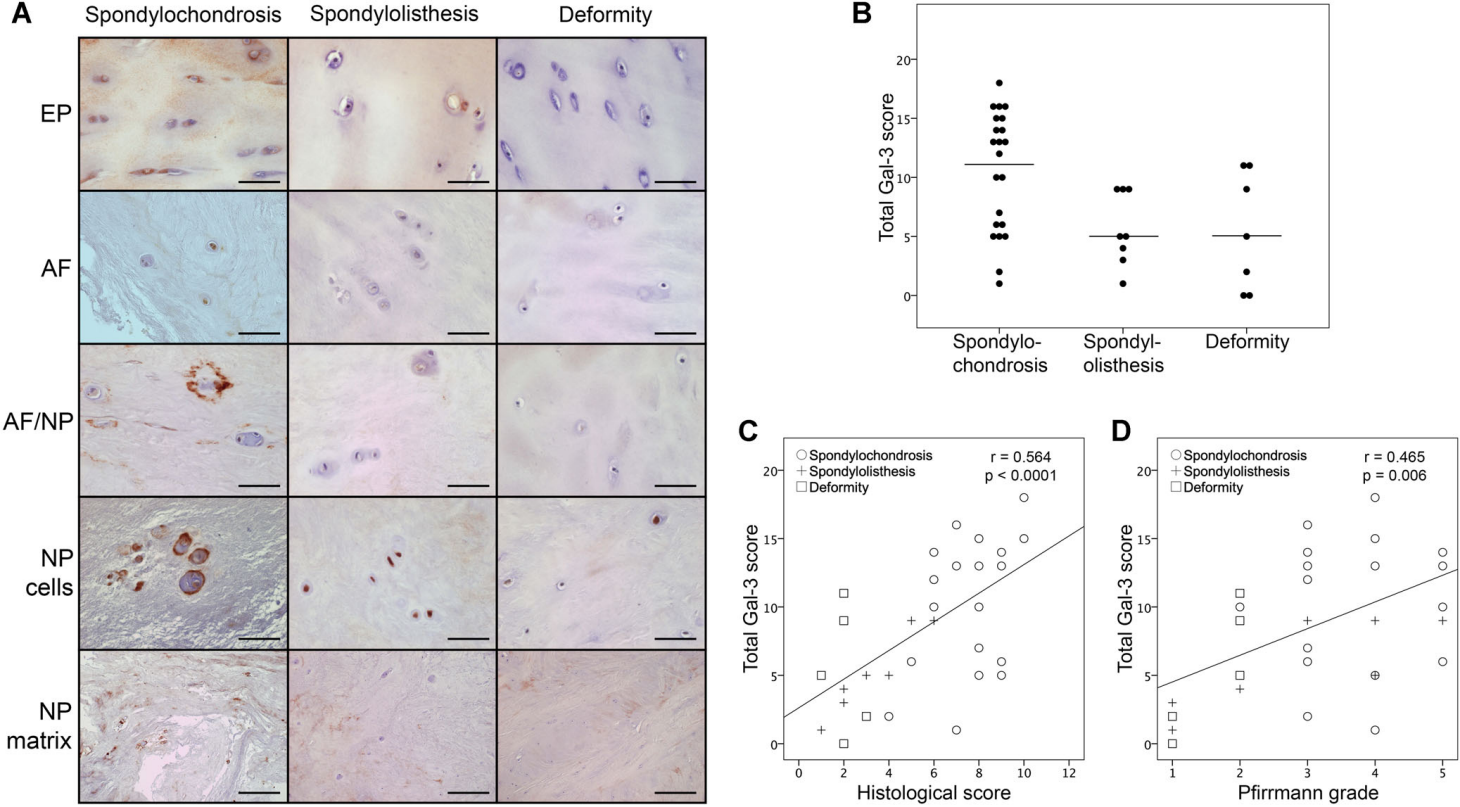
Immunohistochemically detected presence of Gal‐3 in clinical intervertebral disc (IVD) specimens and its correlation with clinical and histological signs of degeneration. (A) Shown are representative IVD specimens from patients of the spondylochondrosis (left column), the spondylolisthesis (middle column), and the deformity cohorts (right column). Gal‐3 positivity (brown color) in the endplate (EP), the anulus fibrosus (AF), the boundary region between AF and NP (AF/NP), as well as for cells or matrix of the nucleus pulposus (NP) is presented. Scale bars = 50 or 200 µm (NP matrix). (B) Comparison of total Gal‐3 LI scores between the spondylochondrosis, spondylolisthesis, and deformity cohorts. Results are presented as dotplots showing Gal‐3 LI scores for patients of each cohort. The median values are indicated as bars. *p < 0.05 (Kruskal–Wallis test). (C,D) Shown are scatterplots of total Gal‐3 LI scores versus (C) histological IVD scores or (D) Pfirrmann grades for patients of the three cohorts with the regression line averaged over all patients. The Spearman correlation coefficient *r* and the *p* value (bivariate correlation test) are given. [Color figure can be viewed at wileyonlinelibrary.com]

Figure 3A illustrates immunopositivity for Gal‐3 in histological IVD sections from representative patients of the three cohorts. Similarly to Gal‐1, Gal‐3 was present in the four main sites, with a tendency for comparatively intense staining in the pericellular matrix (Fig. 2). The medians of total Gal‐3 LI scores (±IQ range) were 12 ± 10 in the spondylochondrosis cohort, 5 ± 5.57 in the spondylolisthesis cohort and 5 ± 11 in the deformity cohort.

Within the spondylochondrosis specimens, the level of Gal‐3 positivity was higher in NP cells than in EP cells and NP matrix (*p *= 0.008 and *p *= 0.03, respectively; Table 3). However, there were no significant differences in Gal‐3 positivity across the different IVD components within spondylolisthesis or deformity specimens (*p* > 0.05; Table 3).

**Table 3:** Immunohistochemical Scores of Gal‐3 Positivity

	Spondylochondrosis (Median ± IQR)	Spondylolisthesis (Median ± IQR)	Deformity (Median ± IQR)	Kruskal–Wallis Test	Overall Median ± IQR
EP	1 ± 1	1 ± 1	1 ± 1	>0.05^a^	1 ± 1
				>0.05^b^	
				>0.05^c^	
AF	3 ± 3	1 ± 1	1 ± 3	>0.05^a^	2 ± 2
				>0.05^b^	
				>0.05^c^	
AF/NP	2 ± 3	1 ± 0.75	1 ± 3	>0.05^a^	1 ± 2
				>0.05^b^	
				>0.05^c^	
NP cells	3 ± 3	1 ± 1.75	1 ± 3	0.02^a^	3 ± 2
				>0.05^b^	
				>0.05^c^	
NP matrix	1 ± 2	1 ± 1	1 ± 1	>0.05^a^	1 ± 1
				>0.05^b^	
				>0.05^c^	
Total Gal‐3 score	12 ± 10	5 ± 5.75	5 ± 11	>0.05^a^	9 ± 8
				>0.05^b^	
				>0.05^c^	
Friedman test (pairwise comparison)	*p* = 0.008 (EP vs. NP cells)				*p* = 0.018 (EP vs. AF/NP)
	*p* = 0.03 (NP cells vs. NP matrix)				*p *< 0.008 (EP vs. AF)
					*p* < 0.0001 (EP vs. NP cells)
					*p *= 0.029 (NP matrix vs. NP cells)

AF, anulus fibrosus; EP, endplate; IQR, interquartile range; IVD, intervertebral disc; NP, nucleus pulposus.

Shown are the median values (±IQR) of Gal‐3 positivity scores in EP, AF, AF/NP, and NP cells and in NP matrix as well as the total Gal‐3 positivity scores for all three cohorts. In addition, *p* values of Kruskal–Wallis test between the three cohorts as well as *p* values of the Friedman test (with pairwise comparison and Bonferroni correction) between the different components of the IVD within a cohort are presented.

^a^ Spondylochondrosis versus spondylolisthesis.

^b^ Spondylochondrosis versus deformity.

^c^ Spondylolisthesis versus deformity.

Comparison of total Gal‐3 LI scores across the three study groups revealed no statistically significant difference (*p* > 0.05; Fig. 3B and Table 3). However, as presented in Table 3, Gal‐3 positivity of NP cells in spondylochondrosis specimens was significantly higher than that of NP cells in spondylolisthesis specimens (*p *= 0.02).

Further correlation analyses of all specimens, irrespective of the assigned study group, revealed a moderate degree of correlation of total Gal‐3 LI scores with historical scores (*r* = 0.564, *p *< 0.0001; Fig. 3C) or the Pfirrmann grades (*r* = 0.465, *p* = 0.006; Fig. 3D), respectively.

### 3.4 Correlation Analyses for Age and Total Gal‐1/Gal‐3 Scores

**Figure 4:**
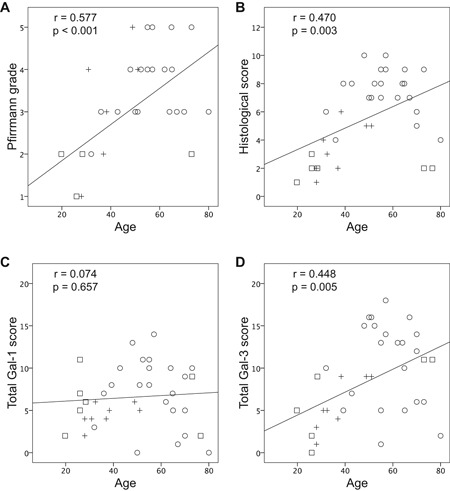
Correlation of clinical and immunohistochemical scores with age of patients. Shown are scatterplots of the patients’ age (from the three cohorts) versus (A) Pfirrmann grades, (B) histological IVD scores, (C) total Gal‐1 LI scores, and (D) total Gal‐3 LI scores. Regression lines, Spearman coefficients (*r*) and *p* values are provided.

**Figure 5:**
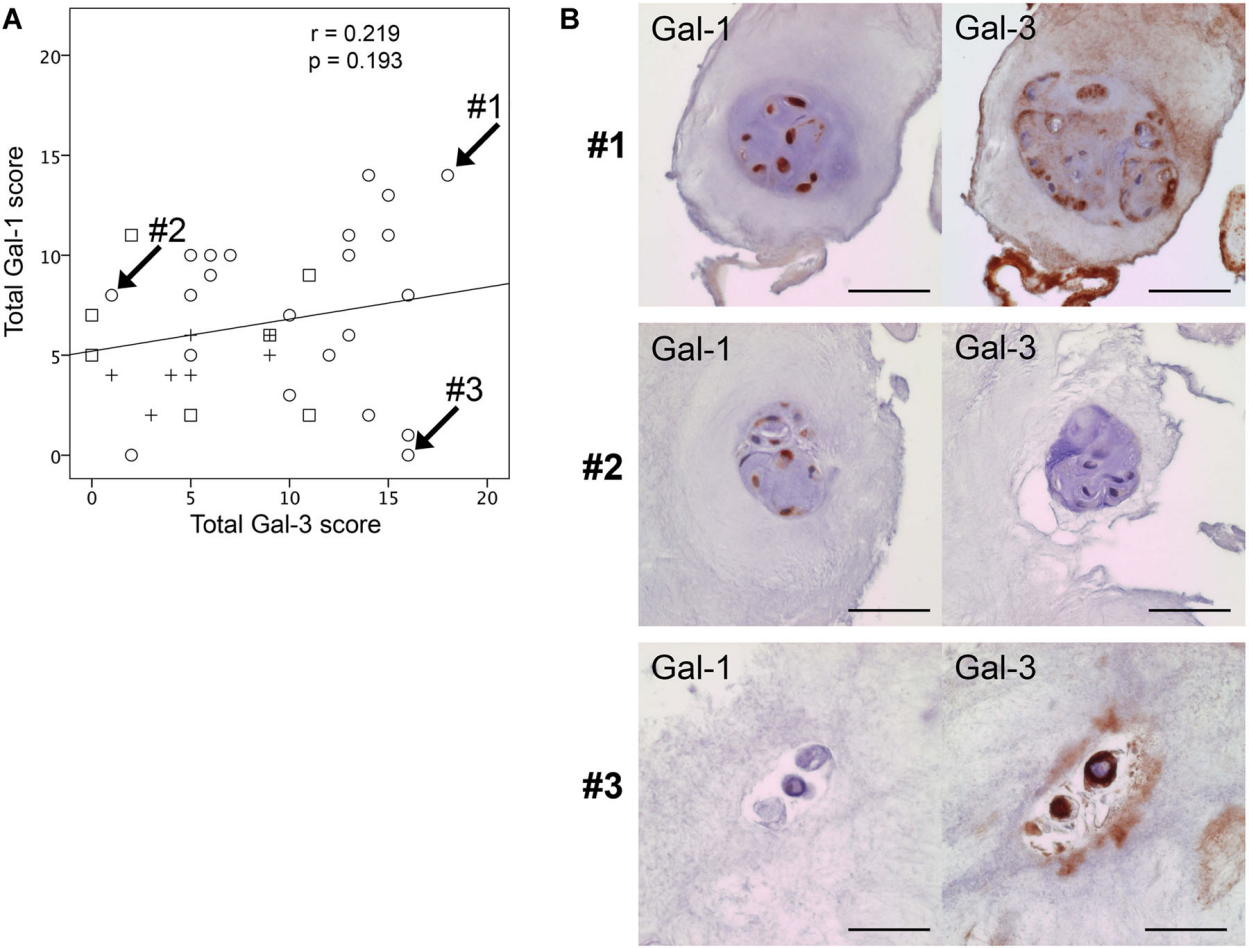
Scores of immunopositivity and patterns of localization of Gal‐1 and Gal‐3 do not correlate in clinical intervertebral disc (IVD) specimens. (A) Scatterplot of total Gal‐1 LI scores versus total Gal‐3 LI scores for patients of the three cohorts with the regression line. Values for the Spearman coefficients (*r*) and *p* values are provided. #1, #2, and #3 are representative specimens with different percentage of positivity for Gal‐1 and Gal‐3. (B) Consecutive histological sections of the three selected IVD specimens were processed immunohistochemically using antibodies against Gal‐1 or Gal‐3, followed by microscopic evaluation of nucleus pulposus cells. #1 shows 75–100% positivity for both Gal‐1 and Gal‐3. #2 shows 75–100% positivity for Gal‐1 and <25% positivity for Gal‐3. #3 shows <25% positivity for Gal‐1 and 75–100% positivity for Gal‐3. Scale bars = 50 µm. [Color figure can be viewed at wileyonlinelibrary.com]

Analyses revealed a moderate degree of correlation of age with the Pfirrmann grade (*r* = 0.577, *p *< 0.001; Fig. 4A), the historical score (*r *= 0.470, *p* = 0.003; Fig. 4B), and the total Gal‐3 LI score (*r* = 0.448, *p *= 0.005; Fig. 4D). In contrast, however, there was no significant correlation between age and the total Gal‐1 LI score (*r* = 0.074, *p *> 0.05; Fig. 4C). Of note, there was also no marked correlation between the total LI scores of Gal‐1 and Gal‐3 (Fig. 5A). Figure 5B shows representative specimens of donors with different level of positivity for Gal‐1 and for Gal‐3, as documented in Figure 5A. Processing serial sections of the three selected IVD specimens immunohistochemically with solutions containing antibodies against Gal‐1 or Gal‐3, respectively, and performing microscopic evaluation of NP cells, variability of staining profiles was observed in pairwise comparison. Shown are representative specimens with different extents of immunohistochemical cell labeling, that is, with 75–100% positivity for both Gal‐1 and Gal‐3, with 75–100% positivity for Gal‐1 and <25% positivity for Gal‐3 and with <25% positivity for Gal‐1 and 75–100% positivity for Gal‐3 (Fig. 5B). Together, these panels suggest that the extent of positivity of NP cells for these two galectins is not strictly coregulated in the degenerated IVD.

### 3.5 Galectin‐Mediated Effects on Functional Disease Markers in Isolated IVD Cells

**Figure 6:**
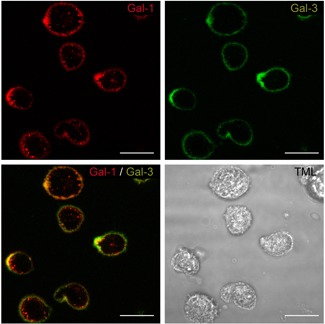
Localization of binding sites for labeled Gal‐1 and ‐3 in isolated intervertebral disc (IVD) cells in vitro. Resuspended IVD cells in passage 1 were labeled with Gal‐1‐AlexaFluor555 (red; left) and Gal‐3‐AlexaFluor488 (green; middle) and analyzed using laser scanning microscopy, with the focus plane set to the center of cells (transmitted light (TML) image: right). Shown are the staining profiles of IVD cells of one patient, representative for experiments with cells obtained from a total of four donors, for each galectin and after signal merging. Scale bars = 20 µm. [Color figure can be viewed at wileyonlinelibrary.com]

First, qPCR analysis detected LGALS1‐ and LGALS3‐specific mRNAs (encoding Gal‐1 and Gal‐3, respectively) in isolated IVD cells at passage 1. LGALS1 (19.4 ± 9.3 molecules/molecules SDHA) was expressed at significantly higher levels than LGALS3 (7.3 ± 0.8 molecules/molecules SDHA, *p* < 0.05; Wilcoxon test; *n* = 5 discs from four patients). In agreement, Gal‐1 and −3 were found in supernatants of IVD cells at concentrations of 10.5 ± 3.7 and 1.0 ± 0.4 ng/ml, respectively (*n *= 8 discs from six patients; *p* < 0.05, Wilcoxon test). Thus, IVD cells actively secrete these two galectins into the medium, where they can act as auto‐ and/or paracrine factors, if capable to bind to the cell surface. To test for galectin binding, fluorescent galectins were used, allowing two‐color staining. When applying a mixture of labeled Gal‐1 and ‐3 on viable IVD cells at 4°C (*n* = 4 patients), strong staining of cellular membranes was observed (Fig. 6). Presence of cognate sugar (lactose) precluded binding (not shown).

**Figure 7:**
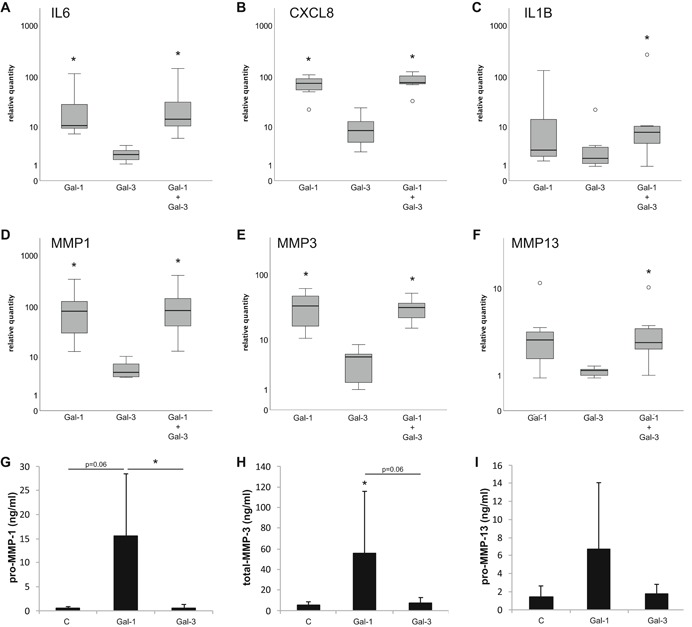
Effects of Gal‐1 and Gal‐3 on the expression of marker genes for intervertebral disc (IVD) degeneration. IVD cells in passage 1 were treated with 10 µg/ml Gal‐1 and/or 18 µg/ml Gal‐3 for 24 h. Messenger RNA (mRNA) levels of interleukin 6 (IL6) (A), CXCL8 (B), IL1B (C), MMP1 (D), MMP3 (E), and MMP13 (F) were determined using quantitative reverse‐transcription polymerase chain reaction (RT‐qPCR) (*n*  =  7 discs from five patients). Results are expressed as relative quantities (boxplots) on a logarithmic scale compared to untreated controls set to 1. (G–I) Concentrations (ng/ml) of pro‐MMP‐1 (G), total‐MMP‐3 (H), and pro‐MMP‐13 (I) in cell culture supernatants of IVD cells (*n* = 6 discs from six patients) treated with 10 µg/ml or 18 µg/ml Gal‐3 for 24 h, as determined by enzyme‐linked immunosorbent assay (ELISA). *p  <  0.05 (Friedman test with pairwise comparison and Bonferroni correction).

Aiming to probe into post‐binding signaling, qPCR assays identified Gal‐1 as a potent inducer of functional disease markers in IVD cells (Fig. 7). In detail, IL6 (median ± IQR: 10.6 ± 26.6‐fold, *p* = 0.006; Fig. 7A), CXCL8 (75.0 ± 46.8‐fold, *p* = 0.011; Fig. 7B), IL1B (2.9 ± 36.8‐fold, *p *= 0.058; Fig. 7C) as well as MMP1 (82.3 ± 145.4‐fold, *p *= 0.011; Fig. 7D), MMP3 (33.7 ± 39.7‐fold, *p* = 0.004; Fig. 7E) and MMP13 (3.0 ± 2.7‐fold, *p* = 0.137; Fig. 7F) were upregulated after exposure to 10 µg/ml Gal‐1 in seven IVD cell populations isolated from five patients. In contrast, Gal‐3 (18 µg/ml; equimolar in monomeric units) did not cause significant effects on any of the analyzed marker genes in the same cell populations (Fig. 7A–F). Increasing the concentration of Gal‐3 to 90 µg/ml did not significantly upregulate these markers (Wilcoxon test, *n* = 3 patients): IL6 (median: 4.5‐fold, *p* = 0.109), CXCL8 (6.9‐fold, *p* = 0.109), IL1B (2.4‐fold, *p* = 0.285) as well as MMP1 (6.8‐fold, *p *= 0.109), MMP3 (4.0‐fold, *p* = 0.285), and MMP13 (1.7‐fold, *p* = 0.109). To investigate the effects of a combined treatment, IVD cells were treated with a mixture of Gal‐1 (10 µg/ml) and Gal‐3 (18 µg/ml). This resulted in upregulation of gene expression comparable to that induced by Gal‐1 alone (Fig. 7A–F). Another common marker for degradation, ADAMTS5, in contrast, was not significantly modified by Gal‐1 and Gal‐3, alone or in combination (data not shown). Preliminary evidence on testing separated AF and NP cell populations of a single patient indicates possibility for differences in relative degree of responsiveness to galectin exposure with respect to type of target gene and cell type (data not shown).

**Figure 8:**
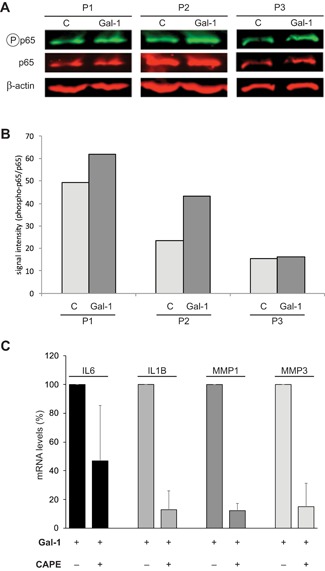
Effects of Gal‐1 and Gal‐3 on the extent of phosphorylation of p65 in intervertebral disc (IVD) cells and nuclear factor‐kB (NF‐kB)‐responsive genes. IVD cells from three patients (P1‐3) in passage 1 were treated with 10 µg/ml Gal‐1 for 15 min. (A) Shown are blots of extracts obtained from three patients for detection of phosphorylated p65, total p65, and β‐actin. (B) Ratios between phosphorylated p65 and total p65 (both normalized to β‐actin), were calculated and depicted as absolute signal intensities. (C) IVD cells (*n* = 2 patients) were treated for 24 h with 10 µg/ml Gal‐1 and 40 µM CAPE. Quantitative reverse‐transcription polymerase chain reaction (RT‐qPCR) analyses of IL6, IL1B, MMP1, and MMP3 messenger RNA (mRNA) levels were performed. The graphs show the percentage of Gal‐1 activity in the absence (equals 100%) and presence of CAPE. [Color figure can be viewed at wileyonlinelibrary.com]

In agreement with qPCR data, ELISAs revealed an induction by Gal‐1 of pro‐MMP‐1, total MMP‐3, and pro‐MMP‐13 secretion in IVD cells, whereas Gal‐3 did not affect the secretion of these markers (Fig. 7G–I). On the basis of previous experience with Gal‐1 and osteoarthritic chondrocytes,^6^ western blot assays were performed to trace involvement of the NF‐kB‐dependent signaling pathway. Figure 8A shows that incubation with 10 µg/ml Gal‐1 for 15 min resulted in a mild increase of p65 phosphorylation in all three tested IVD cell populations isolated from different patients. In quantitative terms, the extent of phospho‐p65 upregulation ranged between 10% and 90% (mean ± SD: 43 ± 42%, *n *= 3), when data were normalized to total p65 and β‐actin as loading control (Fig. 8B). Fittingly, CAPE (an inhibitor of NF‐κB translocation into the nucleus) markedly reduced mRNA levels of the marker genes IL6, IL1B, MMP1, and MMP3 in IVD cells to a range of about 12–47% (Fig. 8C).

## DISCUSSION

The multifunctionality of galectins argues in favor of thorough localization studies in combination with functional testing, to uncover their relevance for disease manifestation. Also, the emerging concept of teamwork among galectins, initially tested in mixtures of Gal‐1 and ‐3 leading to detection of antagonism^38^ or cooperation,^7^ suggests to map expression profiles beyond a single family member. In this report, we characterized the distribution profiles of Gal‐1 and ‐3 in human IVD degeneration. Of note, common absence of a signal peptide explains their intracellular positivity, exemplarily documented in Figure 2. Of clinical relevance, galectins occurred extracellularly in tissue sections and in supernatants of primary cultures, and galectins bound to IVD cells and had a different impact on expression of functional disease markers.

Our study followed a stepwise design. First, we collected clinical IVD specimens of three different spinal diseases. In agreement with previous reports,^39^, ^40^ the histopathological degeneration scores of the IVD specimens significantly correlated with MRI‐based grading, thus supporting the eligibility of the included clinical specimens and the histological scoring. Our results further revealed that proteoglycan content (SO staining) and NP cells were generally more affected by disease progression than the EP, the NP matrix, the AF, and—in terms of the proteoglycan content—than the AF/NP boundary.

Having thus obtained an overview of the clinical material, the immunohistochemical analyses revealed positivity for Gal‐1 in NP (both cells and matrix), the AF and the AF/NP boundary. Matrix positivity is (patho)physiologically relevant, because presentation of Gal‐1 by a mixture of extracellular matrix components (Matrigel) decreased the required quantity for its T cell death‐inducing capacity by one order of magnitude as compared to Gal‐1 in solution.^41^ Whereas chondrocytes of the EP were rarely positive for Gal‐1, comparable extent of Gal‐1 presence was found in cells of the AF and NP. Our data thus extend a previous report on Gal‐1 localization in IVDs of lumbar spine of a human adult with no spinal pathology (age: 35 years),^25^ by considering degenerated tissues and a comprehensive analysis of all IVD compartments.

By adding data on Gal‐3 localization, we further initiated the monitoring of more than one galectin in specimens of degenerated IVDs. Immunopositivity for Gal‐3 was detected in the NP, and also in the other compartments. Its presence within the cells and in the matrix supports the concept of site‐specific activities, for example via its anti‐apoptotic intracellular and glycan‐dependent extracellular mechanisms involving Gal‐3‐mediated counterreceptor aggregation.^42^ A respective impact on chondrocyte survival was inferred by localization studies considering hypertrophy and in a knock‐out model, as also suggested for Gal‐3 in osteoarthritis.^7^, ^43^, ^44^ In rat NP cells, Gal‐3 knock‐down led to increased susceptibility to FasL‐induced cell death,^28^ and a functional cooperation between tumor necrosis factor‐α and Gal‐3 on IL‐1β, IL‐6, and CCL2 expression had been reported.^29^ Considering initial evidence for functional networking among galectins, it becomes reasonable that upcoming studies proceed with the immunohistochemical analysis of further members of this lectin family (such as Gal‐8), at best reaching the level of comprehensive fingerprinting.^45^, ^46^, ^47^, ^48^

When setting the distribution profiles of Gal‐1 and ‐3 in relation to tissue degradation, the total scores of immunopositivity significantly correlated with histopathological degeneration and MRI grading. At the level of IVD components, immunopositivity for Gal‐1 and ‐3 at all sites, except for EP and the NP matrix in the case of Gal‐1, independently correlated with total histopathological degeneration scores of the specimens. However, although histopathological degeneration (and partly MRI scores) differed between the three cohorts, immunohistochemical staining scores for Gal‐1 and ‐3 were similar.

Age is a critical factor for the degeneration of the IVD, both in terms of tissue structure and biochemical composition, as also reflected by our collection of specimens. Concerning the studied galectins, Gal‐3 presence significantly correlated with age. Age‐related changes of Gal‐3 expression have already been documented in the developing human vertebral column^17^ and in a comparison of NP cells between young (29–39 years) and mature (40–59 years) adults.^23^ In contrast, Gal‐1 expression—that significantly correlated with histopathological and MRI‐based evidence for degeneration—did not correlate with patient age. This observation points to the possibility that Gal‐1 may qualify as functional disease marker, warranting efforts to design and test highly specific antagonists such as the recently reported Gal‐3‐like Gal‐1 variant.^49^

As illustrated by work in vitro, Gal‐1 was secreted by IVD cells, bound to their surface and proved active as elicitor of functional disease markers. In contrast, tested in parallel or as part of a mixture (at concentrations previously defined to be active for osteoarthritic chondrocytes^7^, ^8^), Gal‐3 failed to induce expression of relevant genes, thus revealing functional divergence between these two galectins. In agreement, Gal‐1's immunopositivity profile in serial sections did not consistently match Gal‐3 distribution, further arguing against a functional similarity as observed in osteoarthritic chondrocytes.^7^

In summary, our study characterized the distribution profiles of Gal‐1 and ‐3 in the degenerated IVD, thereby establishing the basis for further galectin monitoring in IVD degeneration and supporting the concept of sugar code‐driven mechanisms in common diseases.^10^ Cell‐based experiments describe functional divergence between these two galectins, highlighting homodimeric Gal‐1 as stimulator of disease marker expression, likely via NF‐kB signaling. Of note, modular architecture and glycan fine‐specificity differ for these two galectins.^50^ The strategy to evaluate galectin expression beyond a single protein gives further work a clear direction, that is, to monitor the presence and function of tandem‐repeat‐type galectins such as Gal‐8 and to examine antagonist potency of newly engineered galectin variants.

## ACKNOWLEDGMENTS

The authors sincerely thank the reviewers for their valuable input as well as Melanie Cezanne, Melanie Schmid, and Ruth Gruebl‐Barabas for excellent technical assistance. Stefan Toegel acknowledges generous funding by the Association for Orthopedic Research (AFOR) Foundation.

## Supporting information

Supporting information.

Supporting information.

## References

[1] Andersson GB . 1999 Epidemiological features of chronic low‐back pain. Lancet 354:581–585. 10470716 10.1016/S0140-6736(99)01312-4

[2] Taylor VM , Deyo RA , Cherkin DC , Kreuter W. 1994 Low back pain hospitalization. Recent United States trends and regional variations. Spine 19:1207–1212. 8073311 10.1097/00007632-199405310-00002

[3] Dowdell J , Erwin M , Choma T , et al. 2017 Intervertebral disk degeneration and repair. Neurosurgery 80:S46–S54. 28350945 10.1093/neuros/nyw078 PMC5585783

[4] Sive JI , Baird P , Jeziorsk M , et al. 2002 Expression of chondrocyte markers by cells of normal and degenerate intervertebral discs. Mol Pathol 55:91–97. 11950957 10.1136/mp.55.2.91 PMC1187156

[5] Toegel S , Bieder D , André S , et al. 2014 Human osteoarthritic knee cartilage: fingerprinting of adhesion/growth‐regulatory galectins in vitro and in situ indicates differential upregulation in severe degeneration. Histochem Cell Biol 142:373–388. 24981556 10.1007/s00418-014-1234-x

[6] Toegel S , Weinmann D , André S , et al. 2016 Galectin‐1 couples glycobiology to inflammation in osteoarthritis through the activation of an NF‐κB‐regulated gene network. J Immunol 196:1910–1921. 26792806 10.4049/jimmunol.1501165

[7] Weinmann D , Schlangen K , André S , et al. 2016 Galectin‐3 induces a pro‐degradative/inflammatory gene signature in human chondrocytes, teaming up with galectin‐1 in osteoarthritis pathogenesis. Sci Rep 6:39112. 27982117 10.1038/srep39112 PMC5159921

[8] Weinmann D , Kenn M , Schmidt S , et al. 2018 Galectin‐8 induces functional disease markers in human osteoarthritis and cooperates with galectins‐1 and ‐3. Cell Mol Life Sci 75:4187–4205. 29934665 10.1007/s00018-018-2856-2 PMC6182346

[9] Gabius H‐J , Roth J. 2017 An introduction to the sugar code. Histochem Cell Biol 147:111–117. 27975142 10.1007/s00418-016-1521-9

[10] Manning JC , Romero A , Habermann FA , et al. 2017 Lectins: a primer for histochemists and cell biologists. Histochem Cell Biol 147:199–222. 28013368 10.1007/s00418-016-1524-6

[11] Salamanna F , Veronesi F , Frizziero A , Fini M. 2019 Role and translational implication of galectins in arthritis pathophysiology and treatment: a systematic literature review. J Cell Physiol 234:1588–1605. 30144075 10.1002/jcp.27026

[12] Hu Y , Yéléhé‐Okouma M , Ea H‐K , et al. 2017 Galectin‐3: a key player in arthritis. Joint Bone Spine 84:15–20. 27238188 10.1016/j.jbspin.2016.02.029

[13] Kaltner H , Toegel S , Caballero GG , et al. 2017 Galectins: their network and roles in immunity/tumor growth control. Histochem Cell Biol 147:239–256. 28012132 10.1007/s00418-016-1522-8

[14] Kasai K. 2018 Galectins: quadruple‐faced proteins. Trends Glycosci Glycotechnol 30:SE221–SE223.

[15] Poirier F , Timmons PM , Chan CT , et al. 1992 Expression of the L14 lectin during mouse embryogenesis suggests multiple roles during pre‐ and post‐implantation development. Development 115:143–155. 1638977 10.1242/dev.115.1.143

[16] Fowlis D , Colnot C , Ripoche MA , Poirier F. 1995 Galectin‐3 is expressed in the notochord, developing bones, and skin of the postimplantation mouse embryo. Dev Dyn 203:241–251. 7655085 10.1002/aja.1002030211

[17] Götz W , Kasper M , Miosge N , Hughes RC . 1997 Detection and distribution of the carbohydrate binding protein galectin‐3 in human notochord, intervertebral disc and chordoma. Differentiation 62:149–157. 9447709 10.1046/j.1432-0436.1997.6230149.x

[18] van den Brûle FA , Fernandez PL , Buicu C , et al. 1997 Differential expression of galectin‐1 and galectin‐3 during first trimester human embryogenesis. Dev Dyn 209:399–405. 9264263 10.1002/(SICI)1097-0177(199708)209:4<399::AID-AJA7>3.0.CO;2-D

[19] Hunter CJ , Matyas JR , Duncan NA . 2003 The notochordal cell in the nucleus pulposus: a review in the context of tissue engineering. Tissue Eng 9:667–677. 13678445 10.1089/107632703768247368

[20] Oguz E , Tsai T‐T , Di Martino A , et al. 2007 Galectin‐3 expression in the intervertebral disc: a useful marker of the notochord phenotype? Spine 32:9–16. 17202886 10.1097/01.brs.0000250302.74574.98

[21] Weiler C , Nerlich AG , Schaaf R , et al. 2010 Immunohistochemical identification of notochordal markers in cells in the aging human lumbar intervertebral disc. Eur Spine J 19:1761–1770. 20372940 10.1007/s00586-010-1392-z PMC2989227

[22] Hirata H , Yurube T , Kakutani K , et al. 2014 A rat tail temporary static compression model reproduces different stages of intervertebral disc degeneration with decreased notochordal cell phenotype. J Orthop Res 32:455–463. 24285589 10.1002/jor.22533

[23] Richardson SM , Ludwinski FE , Gnanalingham KK , et al. 2017 Notochordal and nucleus pulposus marker expression is maintained by sub‐populations of adult human nucleus pulposus cells through aging and degeneration. Sci Rep 7:1356. 28473691 10.1038/s41598-017-01567-w PMC5431421

[24] Chen J , Jing L , Gilchrist CL , et al. 2009 Expression of laminin isoforms, receptors, and binding proteins unique to nucleus pulposus cells of immature intervertebral disc. Connect Tissue Res 50:294–306. 19863388 PMC2929695

[25] Jing L , So S , Lim SW , et al. 2012 Differential expression of galectin‐1 and its interactions with cells and laminins in the intervertebral disc. J Orthop Res 30:1923–1931. 22692729 10.1002/jor.22158 PMC3561733

[26] Ohannesian DW , Lotan D , Thomas P , et al. 1995 Carcinoembryonic antigen and other glycoconjugates act as ligands for galectin‐3 in human colon carcinoma cells. Cancer Res 55:2191–2199. 7743523

[27] André S , Kojima S , Yamazaki N , et al. 1999 Galectins‐1 and ‐3 and their ligands in tumor biology. Non‐uniform properties in cell‐surface presentation and modulation of adhesion to matrix glycoproteins for various tumor cell lines, in biodistribution of free and liposome‐bound galectins and in their expression by breast and colorectal carcinomas with/without metastatic propensity. J Cancer Res Clin Oncol 125:461–474. 10480338 10.1007/s004320050303 PMC12172401

[28] Zeng Y , Danielson KG , Albert TJ , et al. 2007 HIF‐1 alpha is a regulator of galectin‐3 expression in the intervertebral disc. J Bone Miner Res 22:1851–1861. 17592963 10.1359/jbmr.070620

[29] Tian Y , Yuan W , Li J , et al. 2016 TGFβ regulates galectin‐3 expression through canonical Smad3 signaling pathway in nucleus pulposus cells: implications in intervertebral disc degeneration. Matrix Biol 50:39–52. 26639428 10.1016/j.matbio.2015.11.008 PMC4808357

[30] Risbud MV , Shapiro IM . 2014 Role of cytokines in intervertebral disc degeneration: pain and disc content. Nat Rev Rheumatol 10(1):44–56. 24166242 10.1038/nrrheum.2013.160 PMC4151534

[31] Wang X , Wang H , Yang H , et al. 2014 Tumor necrosis factor‐α‐ and interleukin‐1β‐dependent matrix metalloproteinase‐3 expression in nucleus pulposus cells requires cooperative signaling via syndecan 4 and mitogen‐activated protein kinase‐NF‐κB axis: implications in inflammatory disc disease. Am J Pathol 184:2560–2572. 25063530 10.1016/j.ajpath.2014.06.006 PMC4188173

[32] Kaltner H , Seyrek K , Heck A , et al. 2002 Galectin‐1 and galectin‐3 in fetal development of bovine respiratory and digestive tracts. Comparison of cell type‐specific expression profiles and subcellular localization. Cell Tissue Res 307:35–46. 11810312 10.1007/s004410100457

[33] Pfirrmann CW , Metzdorf A , Zanetti M , et al. 2001 Magnetic resonance classification of lumbar intervertebral disc degeneration. Spine 26:1873–1878. 11568697 10.1097/00007632-200109010-00011

[34] Rutges JPHJ , Duit RA , Kummer JA , et al. 2013 A validated new histological classification for intervertebral disc degeneration. Osteoarthritis Cartilage 21:2039–2047. 24120397 10.1016/j.joca.2013.10.001

[35] Tiaden AN , Klawitter M , Lux V , et al. 2012 Detrimental role for human high temperature requirement serine protease A1 (HTRA1) in the pathogenesis of intervertebral disc (IVD) degeneration. J Biol Chem 287:21335–21345. 22556410 10.1074/jbc.M112.341032 PMC3375554

[36] Toegel S , Bieder D , André S , et al. 2013 Glycophenotyping of osteoarthritic cartilage and chondrocytes by RT‐qPCR, mass spectrometry, histochemistry with plant/human lectins and lectin localization with a glycoprotein. Arthritis Res Ther 15:R147. 24289744 10.1186/ar4330 PMC3978707

[37] Bustin SA , Beaulieu J‐F , Huggett J , et al. 2010 MIQE précis: practical implementation of minimum standard guidelines for fluorescence‐based quantitative real‐time PCR experiments. BMC Mol Biol 11:74. 20858237 10.1186/1471-2199-11-74 PMC2955025

[38] Xiao Q , Ludwig A‐K , Romanò C , et al. 2018 Exploring functional pairing between surface glycoconjugates and human galectins using programmable glycodendrimersomes. Proc Natl Acad Sci USA 116:E2509–E2518. 10.1073/pnas.1720055115 PMC5856548 29382751

[39] Canbay S , Turhan N , Bozkurt M , et al. 2013 Correlation of matrix metalloproteinase‐3 expression with patient age, magnetic resonance imaging and histopathological grade in lumbar disc degeneration. Turk Neurosurg 23:427–433. 24101259 10.5137/1019-5149.JTN.7459-12.0

[40] Zigouris A , Batistatou A , Alexiou GA , et al. 2011 Correlation of matrix metalloproteinases‐1 and ‐3 with patient age and grade of lumbar disc herniation. J Neurosurg Spine 14:268–272. 21184636 10.3171/2010.9.SPINE09935

[41] He J , Baum LG . 2004 Presentation of galectin‐1 by extracellular matrix triggers T cell death. J Biol Chem 279:4705–4712. 14617626 10.1074/jbc.M311183200

[42] Flores‐Ibarra A , Vértesy S , Medrano FJ , et al. 2018 Crystallization of a human galectin‐3 variant with two ordered segments in the shortened N‐terminal tail. Sci Rep 8:9835. 29959397 10.1038/s41598-018-28235-x PMC6026190

[43] Colnot C , Sidhu SS , Balmain N , Poirier F. 2001 Uncoupling of chondrocyte death and vascular invasion in mouse galectin 3 null mutant bones. Dev Biol 229:203–214. 11133164 10.1006/dbio.2000.9933

[44] Guévremont M , Martel‐Pelletier J , Boileau C , et al. 2004 Galectin‐3 surface expression on human adult chondrocytes: a potential substrate for collagenase‐3. Ann Rheum Dis 63:636–643. 15140769 10.1136/ard.2003.007229 PMC1755017

[45] Manning JC , García Caballero G , Knospe C , et al. 2017 Network analysis of adhesion/growth‐regulatory galectins and their binding sites in adult chicken retina and choroid. J Anat 231:23–37. 28425099 10.1111/joa.12612 PMC5472526

[46] Zivicová V , Broz P , Fík Z , et al. 2017 Genome‐wide expression profiling (with focus on the galectin network) in tumor, transition zone and normal tissue of head and neck cancer: marked differences between individual patients and the site of specimen origin. Anticancer Res 37:2275–2288. 28476793 10.21873/anticanres.11565

[47] Manning JC , García Caballero G , Knospe C , et al. 2018 Three‐step monitoring of glycan and galectin profiles in the anterior segment of the adult chicken eye. Ann Anat 217:66–81. 29501632 10.1016/j.aanat.2018.02.002

[48] Manning JC , Caballero GG , Ruiz FM , et al. 2018 Members of the galectin network with deviations from the canonical sequence signature. 2. Galectin‐related protein (GRP). Trends Glycosci Glycotechnol 30:SE11–SE20.

[49] Ludwig A‐K , Michalak M , Xiao Q , et al. 2019 Design‐functionality relationships for adhesion/growth‐regulatory galectins. Proc Natl Acad Sci USA 116:2837–2842. 30718416 10.1073/pnas.1813515116 PMC6386680

[50] Iwaki J , Hirabayashi J. 2018 Carbohydrate‐binding specificity of human galectins: an overview by frontal affinity chromatography. Trends Glycosci Glycotechnol 30:SE137–SE153.

