# Supplementary material for: Galectins‐1 and ‐3 in Human Intervertebral Disc Degeneration: Non‐Uniform Distribution Profiles and Activation of Disease Markers Involving NF‐κB by Galectin‐1
Source: J Orthop Res. 2019 Jun 24;37(10):2204–16. doi: 10.1002/jor.24351 (PMC6771593; doi:10.1002/jor.24351)
Supplement: Supplementary file 1 — Supporting information. [file JOR-37-2204-s001.docx]

**Supplementary Material**

**Supplementary Methods**

**Additional information on clinical specimens**

Surgical IVD specimens were obtained with written consent from patients treated routinely with transforaminal lumbar interbody fusion (TLIF). After exposure of the IVD through midline approach and TLIF access, the initial anulotomy was performed with a No. 11 scalpel. A complete discectomy was carried out including down-pushing curettes, rasps, ring curettes, pituitaries, and Kerrison rongeurs.

The intervertebral spondylochondrosis was diagnosed in patients with decreased intervertebral space, mainly low back pain and irradiating leg pain with or without neurogenic claudication. The spondylolisthesis was diagnosed in patients with low back pain/leg pain with or without neurogenic claudication with slip evident on lateral x-ray. Patients with progressive scoliosis or kyphosis were included in the deformity group. All patients had radiographic assessment of the diagnosis on the weight bearing lumbar AP and lateral neutral view. Mobility and instability were proven on functional x-rays. In MRI, T2 weighted sagittal and axial images were used to detect compression of neurologic elements. Material of patients with degenerative scoliosis, pseudospondylolisthesis, acute infection, hepatitis or HIV and of patients, who refused to sign the informed consent, was excluded from the study.

**Supplementary Table 1.** RT-qPCR checklist with all relevant technical information of RT-qPCR protocols used in the present study.

**Supplementary Table 2. Histological scores.**

Shown are the median values (±IQR) of the histological degeneration scores of EP, AF, AF/NP and NP cells as well as NP matrix, of SO staining of NP matrix, and the total histological score for all three cohorts. In addition, p-values of Kruskal-Wallis test between the three cohorts as well as p-values of the Friedman test (with pairwise comparison and Bonferroni correction) between the different components of the IVD within a cohort are presented. * Spondylochondrosis versus spondylolisthesis; ** spondylochondrosis versus deformity; *** spondylolisthesis versus deformity.

|  | **Spondylochondrosis**  **Median ± IQR** | **Spondylolisthesis**  **Median ± IQR** | **Deformity**  **Median ± IQR** | **Kruskal-Wallis test** | **Overall Median ± IQR** |
| --- | --- | --- | --- | --- | --- |
| **EP** | 1 ± 1 | 0 ± 0 | 0 ± 0 | * > 0.05  ** 0.03  *** > 0.05 | 0 ± 1 |
| **AF** | 1 ± 0 | 0.5 ± 1 | 0 ± 0 | * 0.01  ** < 0.001  *** > 0.05 | 1 ± 1 |
| **AF/NP** | 1 ± 1 | 0.5 ± 1 | 0 ± 1 | * 0.007  ** 0.001  *** > 0.05 | 1 ± 0 |
| **NP cells** | 2 ± 1 | 1 ± 1.5 | 1 ± 1 | * > 0.05  ** 0.03  *** > 0.05 | 1 ± 1 |
| **NP matrix** | 1 ± 0 | 0 ± 1 | 0 ± 0 | * 0.03  ** < 0.001  *** > 0.05 | 1 ± 1 |
| **SO staining**  **(NP matrix)** | 2 ± 0 | 1 ± 0 | 1 ± 0 | * < 0.001  ** < 0.001  *** > 0.05 | 1.5 ± 1 |
| **Total histological score** | 8 ± 3 | 3.5 ± 3 | 2 ± 0 | * 0.002  ** < 0.001  *** > 0.05 | 6 ± 5 |
| **Friedman Test**  **(pairwise comparison)** | p<0.001 (NP cells vs EP)  p<0.001 (SO vs EP)  p=0.002 (SO vs AF)  p=0.002 (SO vs NP matrix)  p=0.014 (EP vs AF/NP) | n.s. | n.s. |  | p<0.0001 (NP cells vs EP)  p=0.019 (NP cells vs AF)  p=0.006 (NP cells vs NP matrix)  p<0.0001 (SO vs EP)  p<0.0001 (SO vs AF)  p<0.0001 (SO vs NP matrix)  p=0.019 (SO vs AF/NP)  p=0.008 (EP vs AF/NP) |

**Supplementary Figure S-1.** Clinical and histological characterization of IVD specimens. (a) T2-weighted sagittal MR images from representative patients of the spondylochondrosis, spondylolisthesis, and deformity cohorts. (left) MRI of a spondylochondrosis patient with Pfirrmann grade V, showing an inhomogeneous structure of the L2-3 disc with hypointense black signal intensity, loss of distinction between the nucleus and the anulus, and with collapse of the disc space. (middle) MRI of a spondylolisthesis patient with Pfirrmann grade III, revealing an inhomogeneous L5-S1 disc structure, an intermediate grey signal intensity, and unclear distinction between nucleus and anulus, the disc height being normal; (right) MRI evidence of a scoliosis patient with Pfirrmann grade I, presenting a homogeneous L4-5 disc structure with a bright hyperintense signal intensity and normal disc height. (b) Histological evaluation of IVD specimens. Shown are HE and SO stainings of histological IVD sections from representative patients of the spondylochondrosis (left column; IVD score: 8), the spondylolisthesis (middle column; IVD score: 3), and the deformity cohorts (right column; IVD score: 2). The endplate (EP), the anulus fibrosus (AF), the boundary region between AF and NP (AF/NP), cells and matrix of the nucleus pulposus (NP), as well as the intensity of SO staining are shown. Scale bars: 200 µm, or 500 µm (SO). (c) Comparison of histological IVD scores between patients of the spondylochondrosis (n=23), spondylolisthesis (n=8) and deformity (n=7) cohorts. Results are presented as dotplots illustrating histological IVD scores for the patients of each cohort. The median values are given as bars. *p<0.05 (Kruskal-Wallis Test). (d) Shown is a scatterplot of histological IVD scores versus Pfirrmann grades for patients of all three cohorts with the regression line. Values for the Spearman correlation coefficient r and the p-value (bivariate correlation test) are given.
