# Supplementary figures and images for: Galectins‐1 and ‐3 in Human Intervertebral Disc Degeneration: Non‐Uniform Distribution Profiles and Activation of Disease Markers Involving NF‐κB by Galectin‐1
Source: J Orthop Res. 2019 Jun 24;37(10):2204–16. doi: 10.1002/jor.24351 (PMC6771593; doi:10.1002/jor.24351)

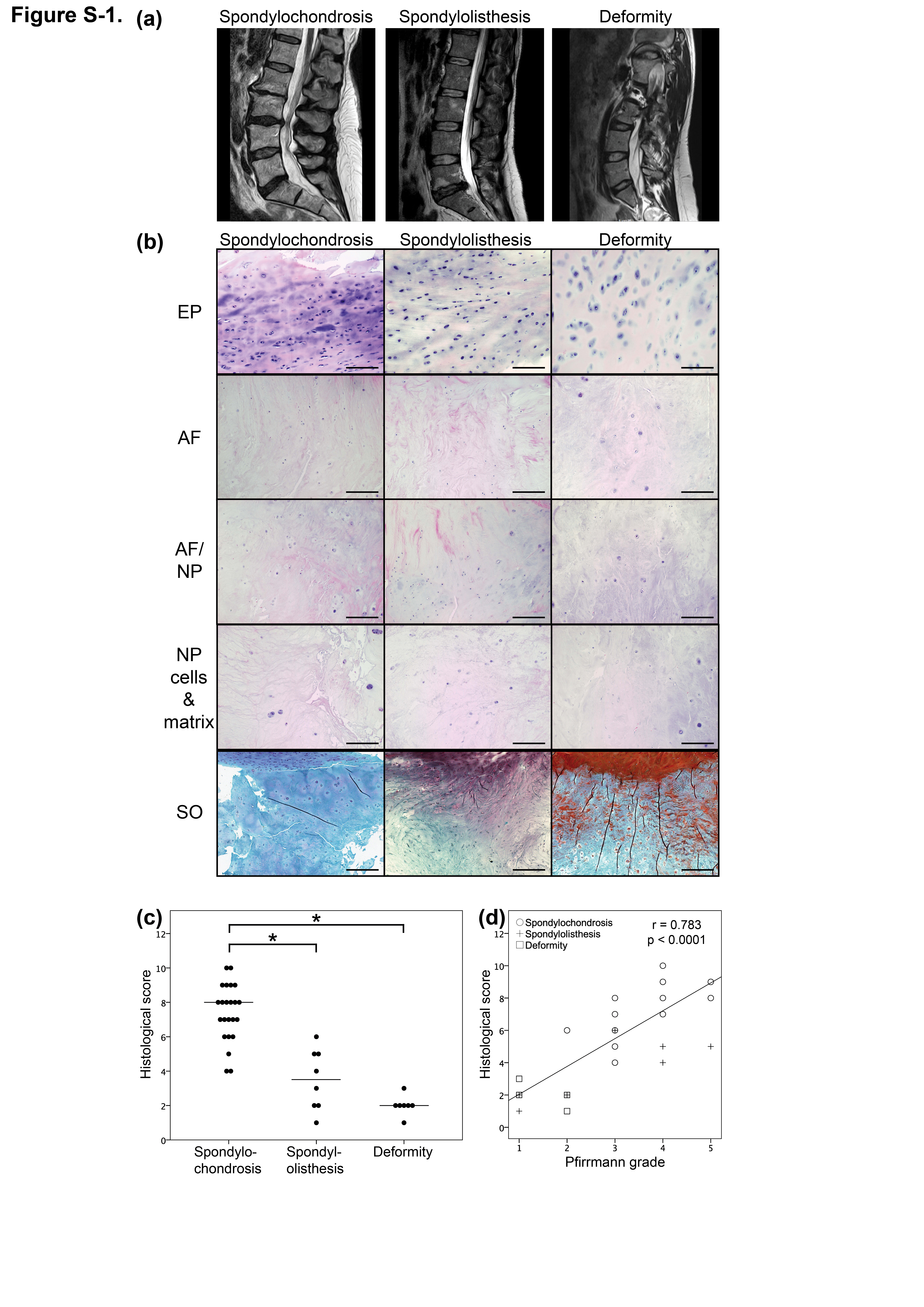

Supplement: Supplementary file 2 — Supporting information. [file JOR-37-2204-s002.jpg]
